# Traditional Chinese Medicine of Angelicae Pubescentis Radix: A Review of Phytochemistry, Pharmacology and Pharmacokinetics

**DOI:** 10.3389/fphar.2020.00335

**Published:** 2020-03-18

**Authors:** Yaqi Lu, Hongwei Wu, Xiankuo Yu, Xiao Zhang, Hanyan Luo, Liying Tang, Zhuju Wang

**Affiliations:** ^1^Institute of Chinese Materia Medica, China Academy of Chinese Medical Science, Beijing, China; ^2^College of Pharmacy, Henan University of Chinese Medicine, Zhengzhou, China

**Keywords:** Angelicae Pubescentis Radix, phytochemistry, pharmacology, pharmacokinetics, botany, traditional medicinal use, toxicity, quality

## Abstract

Angelicae Pubescentis Radix (APR) is a widely used antirheumatic Chinese medicinal herb known as “Duhuo” in China. It has the effects of dispelling wind and removing dampness, diffusing impediment, and relieving pain, and is mainly indicated for rheumatic arthritis with pain in the lower back and knees, and headache. To the best of our knowledge, an attempt is made to provide an up-to-date review on these aspects based on published materials, including ancient and modern books; Master's and doctoral theses; monographs on medicinal plants; the pharmacopoeia of different countries, websites for publication of patent and electronic databases, such as SCI finder, PubMed, Web of Science, ACS, Science Direct, Wiley, Springer, Taylor, CNKI, and Google Scholar. APR, which has a good clinical effect, has been used for traditional Chinese medicine more than 2000 years. Since 1957, a variety of chemical constituents have been reported from the medicinal plants of this herb, mostly coumarins and volatile oil. In the past 30 years, numerous studies have shown that the extracts and compounds isolated from APR showed effective analgesic and anti-inflammatory actions, also showing well effects on central nervous system, effects on cardiovascular system and deworming activity. In addition, we also present and discuss the botany, traditional medicinal use, pharmacokinetics, toxicity, quality control, future trends and prospects of APR. All this information suggest that future research of APR should be supplemented in the area of pharmacology and toxicology to provide further insight on the clinical use and quality control.

## Introduction

Angelicae Pubescentis Radix (equivalent to Angelica Pubescens Root, known as “Duhuo” in China, “Dokwhal” in Korea, “Dokkatsu” in Japan) has been used as herbal medicine extensively since ancient times in Asian countries, including China, Korea and Japan. Due to its effective medicinal value, it has been included in Chinese Pharmacopoeia, British Pharmacopoeia, European Pharmacopoeia, and so on. As an antirheumatic and analgesic agent, it is commonly used as a traditional Chinese medicine (TCM) to treat rheumatic arthralgia and headache. Rheumatic arthralgia is the professional description of TCM syndrome, and includes clinical symptoms such as muscle and joint pain, joint deformities, and dysfunction, with resulting exhaustion and lack of strength. These symptoms are common in rheumatic diseases such as rheumatism and rheumatoid arthritis (RA), with modern medicine ascribing them to an immune deficiency (Wang et al., 2009). In addition, APR is also used in the field of health-care products and cosmetics.

From the Chinese Pharmacopoeia (version 1977), Angelicae Pubescentis Radix (APR) is the dried roots of *Angelica biserrata* (R.H.Shan & C.Q.Yuan) C.Q.Yuan & R.H.Shan (a synonym for *Angelica pubescens* f. *biserrata* R.H. Shan & C.Q. Yuan in Chinese Pharmacopoeia). Besides, *Angelica pubescens* Maxim used to be a plant source of APR in China, and it is still a medicinal plant in Japan. In this review, *A. biserrata* and *A. pubescens* will be reviewed collectively.

APR is mainly produced in Sichuan, Hubei, Anhui and other provinces in China. Usually, its excavation takes place in early spring or late fall. It is common to remove the fibrous roots and sediment, half dried above the heated mud. They were piled for 2 to 3 days, then heated to dry absolutely when they become soft. To date, 87 compounds, including coumarins, polyene-alkynes, phenolic acids, steroids, nucleoside elements have been isolated and identified from APR. What's more, nearly 100 volatile oil compounds have been analyzed by GC-MS. In modern clinical practice, APR plays an important role in treating RA, osteoarthritis pain, vascular dementia (VD), Alzheimer's disease (AD) and headache (Wang et al., 2011a; Jia et al., 2015; Zheng et al., 2017).

In this review, we discussed the botany, traditional medicinal use, phytochemistry, pharmacology, pharmacokinetic, toxicology and quality control of APR as comprehensively as possible, to obtain a comprehensive understanding of the effects of APR and also provide a basis for further research and development of new drugs.

## Botany

*Angelica biserrata* (R.H.Shan & C.Q.Yuan) C.Q.Yuan & R.H.Shan (*A. biserrata*, Chinese name: Chongchi Maodanggui) and *Angelica pubescens* Maxim (*A. pubescens*, Chinese name: Maodanggui) used to be or still be medicinal plants of APR. At present, *A. biserrata* is the plant source of APR recorded in multi-national pharmacopoeia, including Chinese, British, the European. *A. pubescens*, which used to be a medicinal plant for APR in China, is mainly produced in Japan and is used medically in that country at present (Zhengkang, 2006; Rao et al., 1994). In the following discussion, *A. biserrata* and *A. pubescens* will be reviewed collectively.

*A. biserrata* and *A. pubescens* both belong to the genus *Angelica* of the family *Apiaceae*, and the former is a variant of the latter. Due to too much similarity of the plants and constant errors in the study of the resources, some of the *A. biserrata* were once mistaken for *A. pubescens* before the 1990s. According to the resource survey, there is no such a species as *A. pubescens* in China (Institute of Materia Medica, Chinese Academy of Medical Sciences, 1959; Shan et al., 2014). It has been concluded that *A. biserrata* is primarily distributed in China South-Central, China Southeast, and Vietnam, while *A. pubescens* is primarily distributed in Japan and Vietnam (http://www.plantsoftheworldonline.org/).

*A. biserrata* primarily grows on wet and damp slopes, under the forest grass, or in sparse shrubs in Sichuan, Hubei, Jiangxi, Anhui, and Zhejiang provinces, among other selected regions. It is also cultivated in the high mountains of the Sichuan, Hubei, and Shanxi provinces, and breeding methods include seed breeding, direct seeding, and the transplanting of seedlings. In terms of *A. biserrata*, the medicinal material shape of the cultivated variety is similar to the wild product, but the root of the cultivated product is large and soft, with a length of 10–20 cm, and a strong fragrance (http://frps.iplant.cn/; Xiong, 2014).

**Figure 1:**
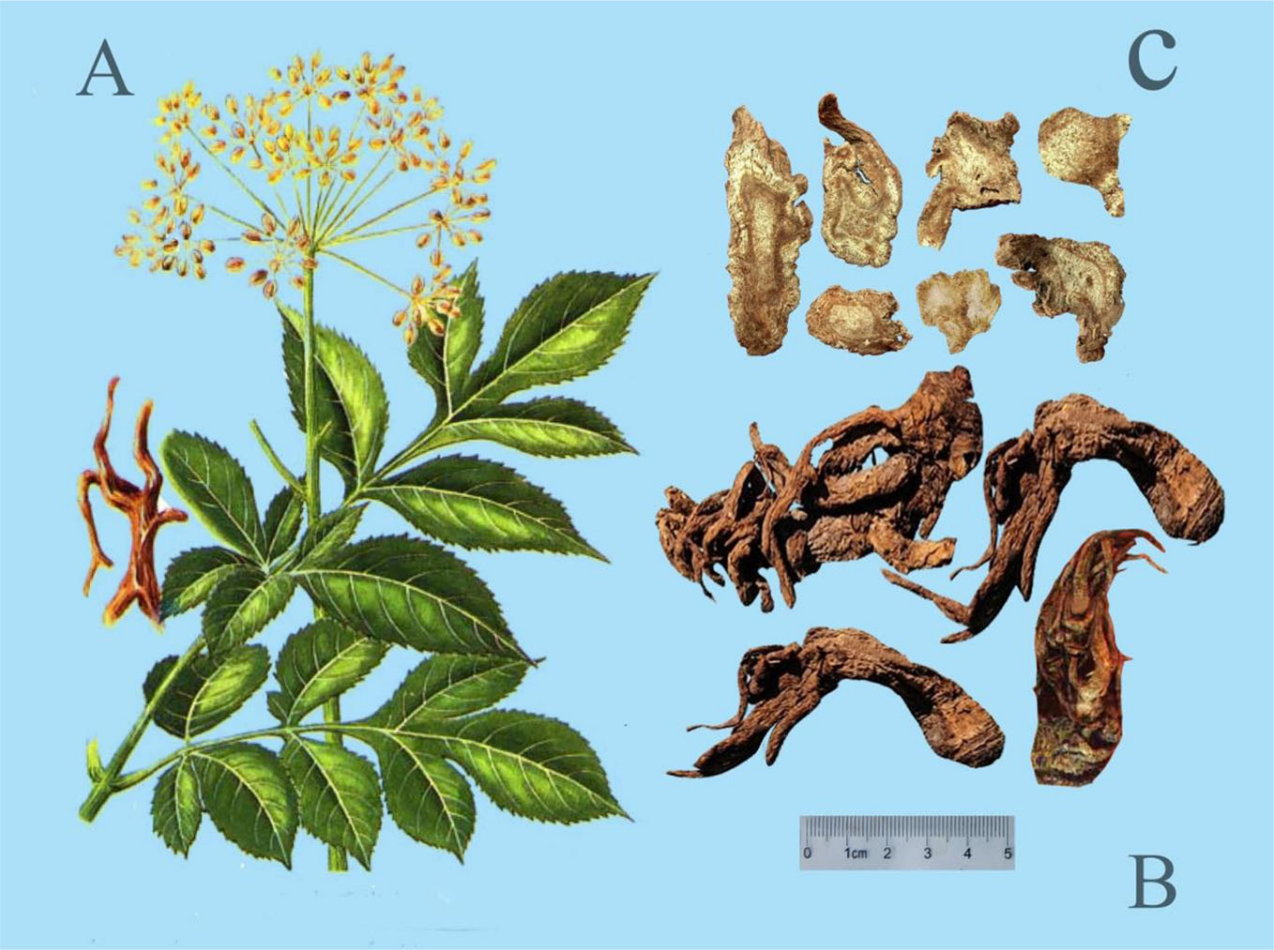
The whole plant of *A. biserrata* **(A)**, medicinal parts of APR **(B)**, the processed of APR **(C)**.

The description of *A. biserrata* and *A. pubescens* plants morphology as referenced by the Flora of China (FOC) and Flora of Japan (FOJ) is presented in **Table 1**. The whole plant of *A. biserrata* (**Figure 1A**), medicinal parts of APR (**Figure 1B**), and the processed of APR (**Figure 1C**) are shown in **Figure 1**. To further distinguish the two plants, the comparison between them were performed based on scientific synonyms included in Kew's taxonomic resources and names published in medicinal references, and the results are shown in **Table 2**.

**Table 1:** The Characteristics and Distribution of medicinal plants as APR.

Scientific name	Chinese name	Characteristics	Distribute	Reference
*Angelica biserrata* (R.H. Shan & C.Q. Yuan) C.Q. Yuan & R.H. Shan/*Angelica pubescens* f. *biserrata* R.H. Shan & C.Q. Yuan	Chongchi Maodanggui重齿毛当归	Plants perennial, 1-2 m, stout. Root cylindric, brown, up to 15 × 1–2.5 cm, aromatic. Stem purplish green, up to 1.5 cm thick, thinly ribbed, hispid above. Basal and lower leaves petiolate, sheaths oblong, inflated, glabrous or slightly pubescent abaxially; blade broad-ovate, 2-ternate-pinnate; leaflets ovate-long-elliptic, base often decurrent along rachis, margin irregularly cuspidate-biserrate, apex acuminate, pubescent along nerves and margin. Peduncles, densely hispidulous; bracts 1, long-subulate, ciliate, deciduous; bracteoles 5–10, broad-lanceolate, apex long-cuspidate, ciliate, pubescent abaxially; umbellules flowered. Calyx teeth obsolete. Petals white, obovate.	Sparse shrubby thickets, damp slopes; Sichuan, Hubei, Jiangxi, Anhui, Zhejiang, Shanxi, Chongqing, Gansu.	FOC^a^ (version 2004)
*Angelica pubescens* Maxim.	Maodanggui毛当归	Stems stout, green, terete, 1–2 m. long, sparingly pubescent; leaves large, about thrice ternately pinnate, the leaflets ovate to elliptic, 5–10 cm. long, sometimes oblong, abrupdy acuminate, often decurrent at base, acutely dentate, pale green beneath, the upper leaves much reduced, with obovate, inflated sheaths; umbels rather numerous, the bracts and bractlets lacking, the rays numerous, 3–16 cm. long, puberulent, the pedicels 25–40, slender. long, puberulent; 2–6 on the commissure, the styles short, shorter than the stylopodium.	Hills and low mountains; Honshu, Shikoku, Kyushu; rather variable.	FOJ^b^ (version 1965)

a Cited from the website: http://foc.iplant.cn/.

b Cited from the website: https://www.ndl.go.jp/.

APR, Angelicae Pubescentis Radix.

**Table 2:** Scientific synonyms and names published in medicinal references of medicinal plants as APR.

Scientific name	No.	Scientific synonyms	No.	Names published in medicinal references
*Angelica biserrata* (R.H. Shan & C.Q. Yuan) C.Q. Yuan & R.H. Shan	1	*Angelica pubescens* f. *biserrata* R.H. Shan & C.Q. Yuan	1	*Angelica biserrata (*R.H. Shan & C.Q. Yuan) C.Q. Yuan & R.H. Shan
			2	*Angelica biserrata* (Shan et Yuan) Yuan et Shan
			3	*Angelica pubescens* Maxim. f. *biserrata* R.H.Shan et C.Q.Yuan
			4	*Angelica pubescens* Maxim. f*. biserrata* Shan et Yuan
*Angelica pubescens* Maxim.	1	*Angelica myriostachys* Koidz.	1	*Angelica pubescens* Maxim.
	2	*Angelica polyclada* Franch.	2	*Angelica pubescens*
	3	*Angelica schishiudo* Koidz.	3	*Angelica polyclada* Franchet
			4	*Angelica pubescens* Maximowicz

Cited from the website: https://mpns.science.kew.org/mpns-portal/.

APR, Angelicae Pubescentis Radix.

## Traditional Medicinal Use

APR is widely used as the important traditional Chinese medicine to treat rheumatic arthralgia and headache for nearly 2000 years. In the Chinese Pharmacopoeia (version 2015), it has been used to treat conditions such as wind-cold-dampness arthralgia, lumbar and knee pain, wind-cold dampness headache, and its recommended dosage is 3–10 g (Committee for the Pharmacopoeia of PR China., 2015).

According to TCM records, APR was initially recorded in “Shen Nong Ben Cao Jing” during the Eastern Han Dynasty (perhaps earlier), which is deemed as the earliest treatise for medicine in China. In this monograph, APR was described as a treatment for wind-cold blows, gold sore pain and hernia mass in women. In “Ben Cao Gang Mu”, another monograph of TCM, APR was described as a treatment to resist stroke, joint pain, tooth swelling and pain by wind. In ancient times, the long-term clinical application was primarily the function of expelling wind and removing dampness, thus relieving pain and exterior syndrome, acting as an antispasmodic, suppressing the hyperactive liver for calming endogenous wind, flattening the Qi, and lowering the inverse to stop vomiting, relieving itching, detoxifying, and inducing hemostasis (Zhang and Liang, 2018).

In addition, APR also has been used with other herbs to treat wind-cold exterior syndrome (TCM term: Fenghan Biaozheng, also can be interpreted as superficial syndrome due to wind-cold), and wind-cold-damp bi-syndromes (TCM term: Fenghanshi Bizheng, also can be interpreted as painful obstructions from wind, damp, and cool environments) in China since ancient times. For example, Duhuo Tang consists of APR together with Angelicae Sinensis Radix, Atractylodis Macrocephalae Rhizoma, Astragali Radix, Cinnamomi Cortex, Achyranthis Bidentatae Radix, and Glycyrrhizae Radix et Rhizoma and can be used to treat rheumatic arthralgia (Gao, 2007). In addition, it was reported that Duhuo Jisheng Tang was effective in treating arthralgia syndrome in the 1990s in Germany (Zuo, 1998). Many classic prescriptions had been created by the ancient famous doctors and have been handed down from generation to generation through repeated clinical verification for thousands of years. Due to its definite clinical effects, TCM prescriptions are the main form of TCM used in clinics. The combination of several different types of TCM can enhance efficacy and reduce adverse reactions.

The application of APR in the field of health care has a long history. Early in “Shen Nong Ben Cao Jing”, there is a record that it can make the body light and slow down your aging. There are also a variety of ways to eat APR, such as Duhuo tea recorded in “Yao Cha Zhi Bai Bing”, Duhuo Renshen wine recorded in “Tai Ping Sheng Hui Fang”, and Duhuo Danggui wine recorded in “Sheng Ji Zong Lu”. In recent years, the application of APR to the field of health care has been further developed, and there have been multiple patent authorizations for inventions, such as a types of APR to dispel wind and dehumidify hot pot material, types of production methods (Wang, 2014), treating a kind of internal injury fever, and APR health wine (Qian, 2014).

In addition to the above application areas, APR has entered the field of beauty and makeup, and has been granted a number of invention patents, such as APR grass oil emollient water (Wang, 2013), and the preparation methods for APR oil tea emulsion (Li, 2013).

## Phytochemistry

**Figure 2:**
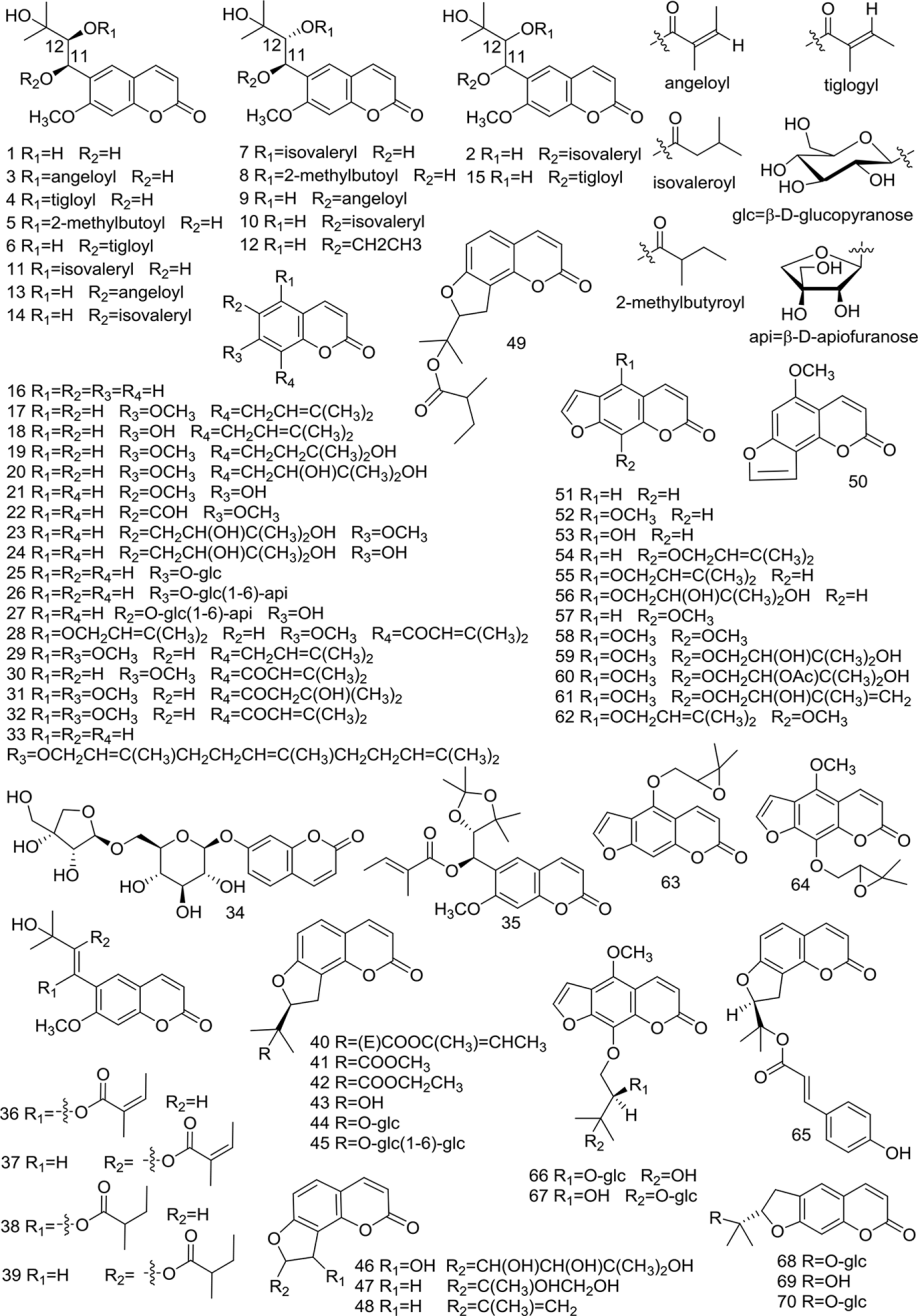
Structures of coumarins **(1–70)** isolated from medicinal plants as APR.

**Figure 3:**
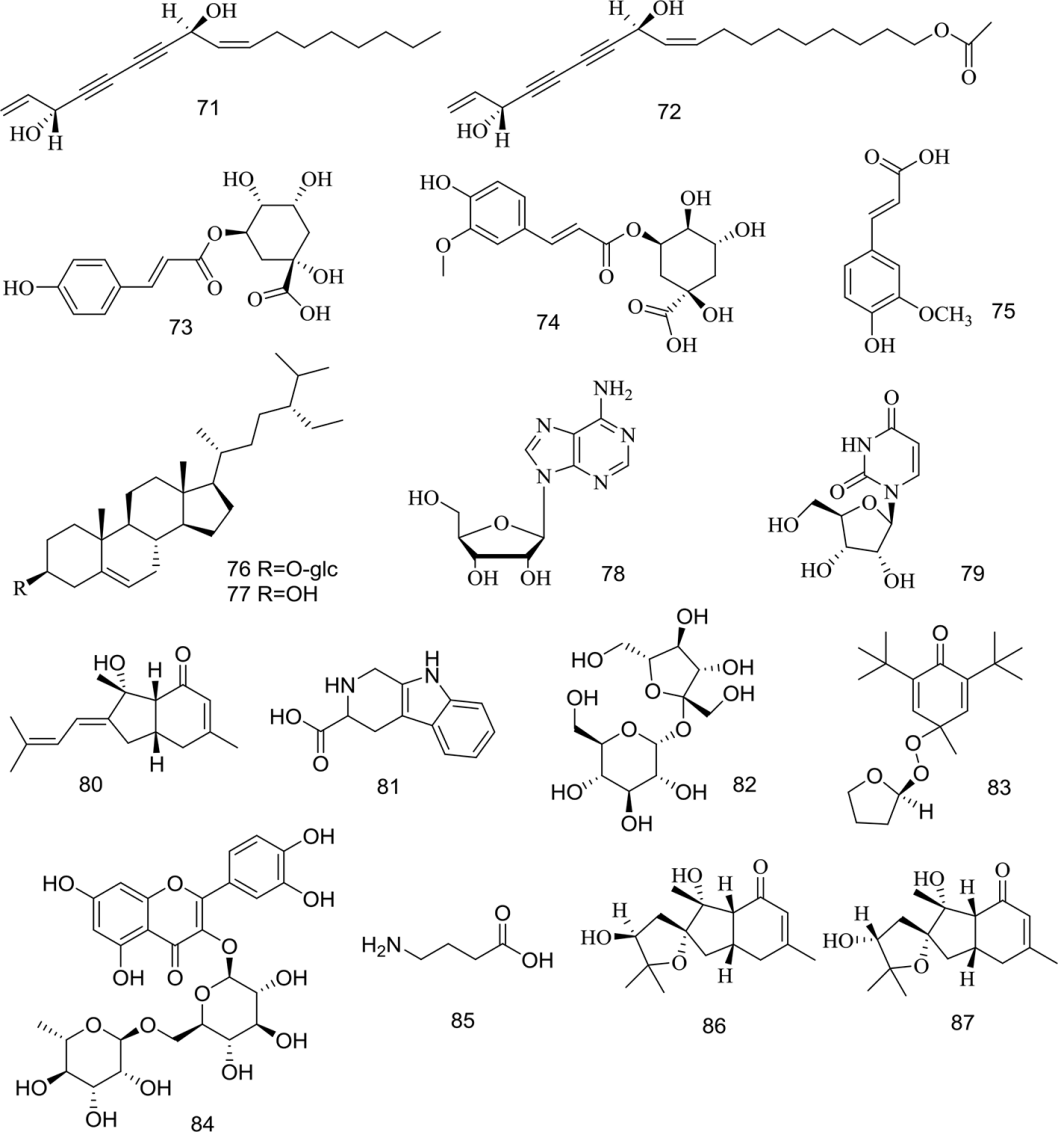
Structures of polyene-alkynes **(71, 72)**, phenolic acids **(73–75)**, steroids **(76–77)** and others **(78–87)** isolated from medicinal plants as APR.

At present, many chemical compounds, including coumarins, polyene-alkynes, phenolic acids, steroids, nucleoside elements, and others, have been isolated and identified from *A. biserrata* and *A. pubescens*. Among these, Coumarins are believed to be the principle non-volatile ingredients with important biological properties. In addition, nearly 100 volatile oil compounds, consisting of terpenoids, small molecular aliphatic and aromatic compounds, have been analyzed by GC-MS (Yang et al., 2006; Wang et al., 2011b). The name, molecular formula, precise mass, and the source of these compounds are listed in **Table 3**. The relevant structures of these compounds are shown in **Figures 2** and **3**.

**Table 3:** Chemical constituents isolated from medicinal plants as APR.

No.	Compound	Molecular Formula	Precise Mass	Source	References
**COUMARINS**					
**1**	Angelitriol	C_15_H_18_O_6_	294.1103	Root & rhizome of *A. biserrata*	Liu et al., 1995a; Liu et al., 1996a
**2**	Anpubesol	C_20_H_26_O_7_	378.1679	Root of *A. biserrata*	Pan et al., 1987
**3**	Angelol A (Angelol)	C_20_H_24_O_7_	376.1522	Root of *A. pubescens*; Root & rhizome of *A. biserrata*	Hata and Kozawa, 1965; Liu et al., 1996b
**4**	Angelol B	C_20_H_24_O_7_	376.1522	Root of *A. pubescens*; Root & rhizome of *A. biserrata*	Baba et al., 1982; Liu et al., 1996b
**5**	Angelol C	C_20_H_26_O_7_	378.1679	Root of *A. pubescens*; Root & rhizome of *A. biserrata*	Baba et al., 1982; Liu et al., 1996b
**6**	Angelol D	C_20_H_24_O_7_	376.1522	Root of *A. pubescens*; Root & rhizome of *A. biserrata*	Baba et al., 1982; Liu et al., 1996b
**7**	Angelol E	C_20_H_26_O_7_	378.1679	Root of *A. pubescens*; Root & rhizome of *A. biserrata*	Baba et al., 1982; Liu et al., 1996b
**8**	Angelol F	C_20_H_26_O_7_	378.1679	Root of *A. pubescens*; Root & rhizome of *A. biserrata*	Baba et al., 1982; Liu et al., 1996b
**9**	Angelol G	C_20_H_24_O_7_	376.1522	Root of *A. pubescens*; Root & rhizome of *A. biserrata*	Baba et al., 1982; Liu et al., 1996b
**10**	Angelol H	C_20_H_26_O_7_	378.1679	Root of *A. pubescens*	Baba et al., 1982
**11**	Angelol I	C_20_H_26_O_7_	378.1679	Root & rhizome of *A. biserrata*	Liu et al., 1996b
**12**	Angelol J	C_17_H_22_O_6_	322.1416	Root & rhizome of *A. biserrata*	Liu et al., 1995a; Liu et al., 1996b
**13**	Angelol K	C_20_H_24_O_7_	376.1522	Root of *A. biserrata*	Liu et al., 1995b; Liu et al., 1996b
**14**	Angelol L	C_20_H_26_O_7_	378.1679	Root of *A. biserrata*	Liu et al., 1995b; Liu et al., 1996b
**15**	Isoangelol	C_24_H_24_O_7_	376.1522	Root of *A. biserrata*	Pan et al., 1987
**16**	Umbelliferone	C_9_H_6_O_3_	162.0317	Fruit of *A. pubescens*; Root & rhizome of *A. biserrata*	Hata et al., 1981; Liu et al., 1994
**17**	Osthole (Osthol)	C_15_H_16_O_3_	244.1099	Fruit of *A. pubescens*; Root of *A. pubescens*	Hata and Kozawa, 1965; Hata et al., 1981
**18**	Osthenol	C_14_H_14_O_3_	230.0943	No mentioned	Wang et al., 2014
**19**	2'-deoxymeranzin hydrate	C_15_H_18_O_4_	262.1205	Root of *A. biserrata*	Zhang et al., 2007
**20**	Meranzin hydrate	C_15_H_18_O_5_	278.1154	Root & rhizome of *A. biserrata*	Liu et al., 1996a
**21**	Scopoletin	C_10_H_8_O_4_	192.0423	Fruit of *A. pubescens*	Hata et al., 1981
**22**	7-methoxy-6-coumarinaldehyde (Angelical)	C_11_H_8_O_4_	204.0423	Root of *A. pubescens*	Hata and Tanaka, 1957
**23**	Ulopterol	C_15_H_18_O_5_	278.1154	Root & rhizome of *A. biserrata*	Liu et al., 1994
**24**	Peucedanol	C_14_H_16_O_5_	264.0988	Root & rhizome of *A. biserrata*	Liu et al., 1994
**25**	7-*O*-*β*-D-glucopyranosyl-umbelliferone	C_15_H_16_O_8_	324.0845	Root & rhizome of *A. biserrata*	Ding et al., 2009
**26**	7-*O*-*β*-D-apiofuranosyl-(1→6)-*β*-D-glucopyranosyl-umbelliferone	C_20_H_24_O_12_	456.1268	Root & rhizome of *A. biserrata*	Ding et al., 2009
**27**	6-*O*-*β*-D-apiofuranosyl-(1→6)-*β*-D-glucopyranosyl-scopoletin	C_20_H_24_O_13_	472.1217	Root & rhizome of *A. biserrata*	Ding et al., 2009
**28**	5-isopentenyloxy-7-methoxy-8-senecioylcoumarin (Angelin)	C_20_H_22_O_5_	342.1467	Root of *A. pubescens*	Kozawa et al., 1980
**29**	Coumurrayin	C_16_H_18_O_4_	274.1205	Root of *A. pubescens*	Kozawa et al., 1980
**30**	7-methoxy-8-senecioylcoumarin	C_15_H_14_O_4_	258.0892	Root of *A. pubescens*	Kozawa et al., 1980
**31**	8-(3-hydroxyisovaleroyl)-5,7-dimethoxycoumarin	C_16_H_18_O_6_	306.1103	Root of *A. pubescens*	Kozawa et al., 1980
**32**	Glabra-lactone	C_16_H_16_O_5_	288.0998	Fruit of *A. pubescens;* Root of *A. pubescens*	Hata and Kozawa, 1965; Hata et al., 1981
**33**	Umbelliprenin	C_24_H_30_O_3_	366.2195	Fruit of *A. pubescens*	Hata et al., 1981
**34**	Apiosylskimmin	C_20_H_24_O_12_	456.1268	Root & rhizome of *A. biserrata*	Liu et al., 1994
**35**	Angepubebisin	C_23_H_28_O _7_	416.1835	Root of *A. biserrata*	Yang et al., 2009
**36**	Isoangelol dehydration	C_20_H_22_O_6_	358.1416	Not mentioned	Wang et al., 2014
**37**	Angelol A dehydration	C_20_H_22_O_6_	358.1416	Not mentioned	Wang et al., 2014
**38**	Anpubesol dehydration	C_20_H_24_O_6_	360.1573	Not mentioned	Wang et al., 2014
**39**	Angelol C dehydration	C_20_H_24_O_6_	360.1573	Not mentioned	Wang et al., 2014
**40**	Columbianadin	C_19_H_20_O_5_	328.1311	Root of *A. biserrata*	Wang et al., 1988
**41**	Columbianetin acetate	C_16_H_16_O_5_	288.0998	Root of *A. biserrata*	Wang et al., 1988
**42**	Columbianetin propionate	C_17_H_8_O_5_	302.1154	Root of *A. biserrata*	Liu et al., 1998a
**43**	Columbianetin	C_14_H_14_0_4_	246.0892	Root of *A. biserrata*	Wang et al., 1988
**44**	Columbianetin-*β*-D-glucopyranoside	C_20_H_24_O_9_	394.1264	Root & rhizome of *A. biserrata*	Li et al., 1989b; Wu and Li, 1993
**45**	Columbianin	C_26_H_34_O_14_	570.1949	Root & rhizome of *A. biserrata*	Liu et al., 1996a
**46**	2'-(1”, 2”, 3”-three hydroxyl)-isoamyl Angelica lactone	C_16_H_18_O_7_	322.1053	Root of *A. biserrata*	Wu and Li, 1993
**47**	Angelidiol(heramandiol)	C_14_H_14_O_5_	262.0841	Root & rhizome of *A. biserrata*	Liu and Yao, 1995; Liu et al., 1996a
**48**	Angenomalin	C_14_H_12_O_3_	228.0786	Root of *A. biserrata*	Yang et al., 2008
**49**	Dihydrocolumbianadin	C_19_H_22_O_5_	330.1467	Root of *A. biserrata*	Wang et al., 2014
**50**	Isobergapten	C_12_H_8_O_4_	216.0423	Root of *A. biserrata*	Sun et al., 2014
**51**	Psoralen	C_11_H_6_O_3_	186.0317	Fruit of *A. pubescens*; Root of *A. biserrata*	Hata et al., 1981; Zhang et al., 2007
**52**	Bergapten	C_12_H_8_O_4_	216.0423	Fruit & root of *A. pubescens*	Hata and Kozawa, 1965; Hata et al., 1981
**53**	Bergaptol	C_11_H_6_O_4_	202.0266	Root of *A. biserrata*	Zhang et al., 2007
**54**	Imperatorin	C_16_H_14_O_4_	270.0892	Root of *A. biserrata*; Fruit of *A. pubescens*	Hata et al., 1981; Sun et al., 2014
**55**	Isoimperatorin	C_16_H_14_O_4_	270.0892	Root of *A. biserrata*	Pan et al., 1987; Wang et al., 2014
**56**	Oxypeucedanin hydrate	C_16_H_16_O_6_	304.0947	Root & rhizome of *A. biserrata*	Liu et al., 1994
**57**	Xanthotoxin	C_12_H_8_O_4_	216.0423	Fruit of *A. pubescens*; Root of *A. biserrata*	Hata et al., 1981; Pan et al., 1987
**58**	Isopimpinellin	C_13_H_10_O_5_	246.0528	Root of *A. pubescens*	Kozawa et al., 1980
**59**	Byak-angelicin	C_17_H_18_O_7_	334.1053	Root of *A. pubescens*	Kozawa et al., 1980
**60**	5-methoxy-8-(2-acethoxy-3-hydroxy-3-methylbutoxy) psoralen [sec-*O*-acetylbyakangelicin]	C_19_H_20_O_8_	376.1158	Fruit of *A. pubescens*	Hata et al., 1981
**61**	Neobyakangelicol	C_17_H_16_O_6_	316.0947	Fruit of *A. pubescens*	Hata et al., 1981
**62**	Cnidilin	C_17_H_16_O_5_	300.0998	Root of *A. biserrata*	Zaugg et al., 2011
**63**	Oxypeucedanin	C_16_H_14_O_5_	286.0841	Fruit of *A. pubescens*	Hata et al., 1981
**64**	Ferulin	C_17_H_16_O_6_	316.0947	Fruit of *A. pubescens*	Hata et al., 1981
**65**	Angelmarin	C_23_H_20_O_6_	392.1260	Rhizome of *A. biserrata*	Awale et al., 2006
**66**	*Sec*-*O*-*β*-D-glucopyranosyl-(R)-byakangelicin	C_23_H_28_O_12_	496.1581	Root of *A. biserrata*	Ding et al., 2008
**67**	*Tert*-*O*-*β*-D-glucopyranosyl-(R)-byakangelicin	C_23_H_28_O_12_	496.1581	Root of *A. biserrata*	Ding et al., 2008
**68**	Marmesinin	C_20_H_24_O_9_	408.1420	Root & rhizome of *A. biserrata*	Liu et al., 1996a
**69**	Nodakenetin	C_14_H_14_O_4_	246.0892	Root & rhizome of *A. biserrata*	Liu et al., 1996a
**70**	Nodakenin	C_20_H_24_O_9_	408.1420	Root & rhizome of *A. biserrata*	Liu et al., 1994
**POLYENE-ALKYNES**					
**71**	Falcarindiol	C_17_H_24_O_2_	260.1776	Root of *A. biserrata*	Liu et al., 1998a
**72**	11(S),16(R)-dihydroxy-octadeca-9Z,17-dien-12,14-diyn-1-yl acetate	C_20_H_28_O_4_	322.1988	Root of *A. biserrata*	Liu et al., 1998a
**PHENOLIC ACIDS**					
**73**	3-*O*-*trans*-coumaroylquinic acid	C_16_H_18_O_8_	338.1002	Root of *A. biserrata*	Yang et al., 2008
**74**	3-*O*-*trans*-feruloylquinic acid	C_17_H_20_O_9_	368.1107	Root of *A. biserrata*	Yang et al., 2008
**75**	Ferulic acid	C_10_H_10_O_4_	194.0579	Root of *A. biserrata*	Sun et al., 2014
**STEROIDS**					
**76**	Daucosterol	C_35_H_60_O_6_	576.4390	Root & rhizome of *A. biserrata*	Liu et al., 1994
**77**	*β*-sitosterol	C_29_H_50_O	414.3862	Root & rhizome of *A. biserrata*	Liu and Yao, 1995
**OTHERS**					
**78**	Adenosine	C_10_H_13_N_5_O_4_	267.0968	Root & rhizome of *A. biserrata*	Liu et al., 1994; Sun et al., 2014
**79**	Uridine	C_9_H_12_N_2_O_6_	243.0632	Root of *A. biserrata*	Sun et al., 2014
**80**	Bisabolangelone	C_15_H_20_O_3_	248.1412	Root of *A. biserrata & A. pubescens*	Liu et al., 1998a
**81**	2,3,4,9-Tetrahydro-1H-pyrido[3,4-b] indole-3-carboxylic acid	C_12_H_12_N_2_O_2_	216.0899	Root & rhizome of *A. biserrata*	Liu et al., 1994
**82**	Sucrose	C_12_H_22_O_11_	342.1162	Root & rhizome of *A. biserrata*	Liu et al., 1994
**83**	Angepubefurin	C_19_H_30_O_4_	322.2144	Root of *A. biserrata*	Yang et al., 2009
**84**	Rutin	C_27_H_30_O_16_	610.1534	Root of *A. biserrata*	Yang, 2007
**85**	*γ*-aminobutyric acid	C_4_H_9_NO_2_	103.0633	Root & rhizome of *A. biserrata*	Li et al., 1989a
**86**	Angesesquid A	C_15_H_22_O_5_	280.1675	Root of *A. biserrata*	Li et al., 2019
**87**	Angesesquid B	C_15_H_22_O_5_	280.1675	Root of *A. biserrata*	Li et al., 2019

APR, Angelicae Pubescentis Radix.

### Coumarins

Coumarins are phenolic compounds characterized by a benzene ring attached to an alpha-pyrone ring, which can be regarded as a lactone formed by the dehydration of o-hydroxy cinnamic acid. Coumarins have a fragrant smell and occur naturally in many plants. In addition, they are known to possess a myriad of pharmacological activities, including antioxidant, anti-cancer, and anticoagulant effects. They are also known to be fluorophores with their fluorescence changing drastically with varying substituents and newly introduced positions. Therefore, coumarins can be identified using fluorescence detection (Murata et al., 2005). So far, the coumarins separated from APR are mainly composed of simple coumarin as the parent nucleus, linear furocoumarin formed by substitution of seven and six positions, and flavonocoumarin formed by substitution of seven and eight positions. Simple coumarins, angular furocoumarins, psoralen furocoumarins, and nodakenetin furocoumarins have been isolated and identified, with the chemical structures of these coumarins shown in **Table 3** and **Figure 2**.

Simple coumarins (**1**–**39**) have one or more substituents at five, six, seven, eight sites in the mother nucleus of coumarin, which are replaced by -OH, -OCH_3_, etc. Some (**25–27**) can be glycosylated with sugar. Structures of the simple coumarins are shown in **Figure 2**.

At present, there are 11 angular furocoumarin (**40**–**50**, **65**) compounds that have been reported. They can be divided into two types, with one type (**40–49**, **65**) having a single bond at the position of 1' and 2', while the other type (**50**) has a double bond. Two (**44**, **45**) of them are glycosylated. Their structures are shown in **Figure 2**.

Currently, 15 psoralen furocoumarins (**51–64**, **66, 67**) have been reported, all of which have the skeleton characteristics of psoralen. The side chains of two compounds (**62**, **64**) have an epoxy structure, the other two (**63**, **64**) are glycosylated, and the remaining 11 compounds are typical psoralen furocoumarins. Their structures are shown in **Figure 2**.

There are reported 3 nodakenetin furocoumarins (**68–70**) currently. The difference in these compounds from the psoralen structure is the hydrogenation of the double bonds on the furan ring. Their structures are shown in **Figure 2**.

### Polyene-Alkynes

Two long chain polyene-alkynes (**71**, **72**) have been isolated from the root of *A. biserrata*, and they have conjugated systems with multiple double bonds and alkyne bonds. Falcarindiol (**71**) has been reported to be an anesthetic with antifungal activity. It also exhibits prominently inhibitory effects on 5-LO with an IC_50_ value of 9.4 μM, and moderate inhibitory activity on COX-1 with an IC_50_ value of 66 μM (Liu et al., 1998a). The chemical structures of the polyene-alkynes are shown in **Figure 3**.

### Phenolic Acids

Three phenolic acids (**73–75**) have been isolated from the root of *A. biserrata*. Since phenolic hydroxyl groups replace their structure, these compounds are unstable and thus easily transformed by water, temperature, light, enzymes, acids, and alkalis (Lin et al., 2017). The chemical structures of the phenolic acids are shown in **Figure 3**.

### Steroids

Two steroids (**76**, **77**) have been isolated from the root and rhizome of *A. biserrata*. They represent fused tetracyclic compounds composed of three hexacyclohexane (A, B, C) and a five-carbon ring (D). In addition, *β*-sitosterol (**77**) is a phytosterol that is common in higher plants. The chemical structures of the steroids are shown in **Figure 3**.

### Other Compounds

Apart from the ingredients above, other compounds have been isolated and identified from *A. biserrata* and *A. pubescens*. Two nucleosides, including adenosine (**78**) and uridine (**79**), have been isolated from the root or rhizome of *A. biserrata*. Bisabolangelone (**80**) has been isolated from *A. pubescens* for the first time with strong anti-feeding properties against insects but it's been reported to be unstable in both basic and acidic media (Liu et al., 1998a). In addition, 2, 3, 4, 9-Tetrahydro-1H-pyrido [3, 4-b] indole-3-carboxylic acid (**81**) and Sucrose (**82**) were isolated from the plant for the first time in 1994 (Liu et al., 1994). A new furan, angepubefurin (**83**), was determined by HR-FTICR-MS and X-ray diffraction analyses (Yang et al., 2009). Rutin (**84**) and *γ*-aminobutyric acid (**85**) have also been isolated and identified in succession (Li et al., 1989a; Yang, 2007). Both new sesquiterpenoid derivatives (**86**) and (**87**) could moderately inhibit the inflammatory reaction. The beneficial elements Ca, Mg, and K in APR have been determined by LIBS spectra and the artificial neural network method (Wang et al., 2018a). The chemical structures of other compounds are shown in **Figure 3**.

## Pharmacology

The study of pharmacological activities has indicated that APR has proven to have positive effects as an analgesic and anti-inflammatory agent, which are important for RA treatment strategies. Other reported functions of APR include effects on the central nervous system, effects on the cardiovascular system, deworming activity. The main pharmacological activities of the extracts or isolated compounds from APR that have been reported in animal and *in vitro* studies are summarized in **Table 4**.

**Table 4:** The pharmacological activities of APR extracts or certain ingredients.

Compounds or extracts	Assay	Effect	Reference
**ANALGESIC AND ANTI-INFLAMMATORY ACTIONS**			
CRAB (1.10 g/ml)	Male SD rats; SNI model of neuropathic pain; 20 mg/kg/d *i.g.*, 14 d	Levels of TNF-*α*, IL-1*β* and IL-6↓, expression of TRPV1 and pERK↓; ID_50_: 10.28 mg/kg	Li et al., 2017
Columbianetin	LPS-stimulated human peripheral blood mononuclear cell model, *in vitro*; 0, 10, 20, 40 μg/ml, 4 h	TNF-*α*, IL-6, MCP-1, and IL-1*β*↓, activity of NOD1, RIP2, and NF-*κβ*↓, NOD1/NF-*κβ* pathways were blocked by ML130	Lu et al., 2018
ERAB (0.82 g/ml)	LPS-induced inflammation model, *in vitro*; 1, 5, 25 mg/L, 6 h	Activity of NAAA↓, PEA↑, TNF-*α*, iNOS and IL-6↓, TNF-*α*, NO↓	Sun et al., 2011
**EFFECTS ON THE CENTRAL NERVOUS SYSTEM**			
WRAB (0.365 g/ml)	Male Wister rats; Rat model of AD induced by intracerebral injection of A*β*_1-40_; 2 ml/d *i.g.*, 28 d	IL-4↑, TNF-*α*↓	Zhu et al., 2009
WRAB	Male Wister rats; Rat model of AD induced by intracerebral injection of A*β*_1-40_; 2 ml/d *i.g.*, 28 d	Expression of p38 mitogen-activated protein kinases↓	Zhang and Du, 2010
WRAB and ARAB (1.10 g/ml)	Male and female Kunming mice; D-galactose brain aging model; 2.7, 8.1, 24.3 g/kg/d *i.g.*, 8 w	Phosphatidylcholine (PC)↑, sphingomyelin (SM)↓, SM/PC↓, IL-2↑	Jin et al., 2003
CRAB (purity 95.8%)	C57BL/6 mice; A*β*-induced neurons damage; 10, 50, 100, 150, 250 mg/ml, 24 h	Expressions of Phosphorylation of CREB and brain-derived neurotrophic factor (BDNF)↑; The optimal dose: 100 mg/ml	Yan et al., 2016
WRAB (1 g/ml)	Male Wister rats; Rat model of VD; 0.3125 ml/100g/d, 28 d	Expressions of MDA↓, SOD↑	Weng et al., 2012
WRAB	Male Wister rats; AD model induced by A*β*_1-40_ protein and VD model of persistent bilateral ligation of bilateral common carotid arteries; 3.125 g/kg/d, 28 d	IL-4↑, TNF-*α*↓	Wang et al., 2011a
RAB extracts of 95% ethanol, petroleum ether, ethyl acetate and petroleum ether-ethyl acetate	SH-SY5Y cells injury induced by hydrogen peroxide, *in vitro*; 10, 50, 100, 250, 500 mg/L, 24 h	SOD↑, MDA↓, level of BDNF and nerve factor-3↑; The optimal dose of APR 95% ethanol and petroleum ether extracts: 500 mg/L, the optimal dose of APR ethyl acetate and petroleum ether-ethyl acetate extracts: 250 mg/L	Hu et al., 2013a
Osthole (> 98% purity)	BM-NSCs against injury induced by hydrogen peroxide lactate dehydrogenase leakage, *in vitro*; 0, 10, 50, and 100 µM, 24h	The expression ratio of Bcl2-associated X/B-cell lymphoma-2 mRNA↓, increase phosphorylation of CREB and PI3K↑	Yan et al., 2017
**THE EFFECT ON THE CARDIOVASCULAR SYSTEM**			
ARAB	MTT, *in vitro*; 3.75, 7.5, 15, 30 μg/ml, 48 h	HUVEC proliferation, migration and tubule formation↓, HUVEC apoptosis↑	Hu et al., 2013b
Osthole	MTT, *in vitro*; 3.75, 7.5, 15, 30 μg/ml, 24 h	HUVEC proliferation, migration and tubule formation↓, HUVEC apoptosis↑	Hu et al., 2013b
Osthole (> 99% purity)	Male Wister rats, *in vitro*; Isolated pulmonary arteries model; 10^-9^ M to 10^-5^ M	NO↑, Akt and eNOS↑, phosphorylations of Akt at Phospho-Akt and eNOS at Phospho-eNOS in endothelial cells↑	Yao et al., 2013

APR, Angelicae Pubescentis Radix.

### Analgesic and Anti-Inflammatory Actions

APR is a high-frequency drug for the treatment of RA, which is an autoimmune disease requiring the use of analgesics and anti-inflammatory medicines to alleviate the associated pain. Some molecules, including extracellular signal-regulated kinase 1/2, arylhydrocarbon receptor, Histone h3, prostaglandin E receptor 2, nuclear factor κappa *β* (NF-*κβ*), programmed cell death 5, interleukin (IL)-36, IL-10, IL-4, Hypoxia Inducible Factor 1A, and arachidonate 15-lipoxygenase, are common molecular targets related to APR and RA, with the Eicosanoid signaling pathway the common pathway of APR and RA (Jia et al., 2015).

In TCM, the crude water extract of the root of *A. biserrata* (RAB) is considered a selective and effective herbal agent in attenuating persistent hindpaw inflammation and hyperalgesia in rats (Wei et al., 1999). RAB consisting of 60% ethanol extract (1.5 g/kg, *i.g.*) significantly inhibits inflammation in three models, including xylene-induced mouse ear edema test, the mice pettitoes swelling by egg white test and mouse tampon granulation swelling test (Li et al., 2013). However, the methanol-, chloroform-, and ethyl acetate extracts from the root of *A. pubescens* not only effectively reduced pain that was induced by 1% acetic acid and a hot plate, but also reduced the edema that was induced by 3% formalin or 1.5% carrageenan (Chen et al., 1995).

The analgesic effect of coumarins isolated from RAB (CRAB, 20 mg/kg, *i.g.*) is mediated by inflammatory factors and transient receptor potential cation channel 1 (TRPV1) as evidenced by a spared nerve injury (SNI) model of neuropathic pain. Molecular profiling has revealed that CRAP reduces levels of the proinflammatory cytokines tumor necrosis factor-*α* (TNF-*α*), IL-1*β*, and IL-6 and significantly attenuates the expressions of TRPV1 and phosphorylated extracellular regulated protein kinases (pERK) in damaged dorsal root ganglion neurons (Li et al., 2017).

Four coumarins (10 mg/kg, *i.p.*), including columbianadin (**40**), columbianetin acetate (**41**), bergapten (**52**) and umbelliferone (**16**), have been demonstrated to have significant anti-inflammatory and analgesic activities. While the coumarins osthole (**17**) and xanthotoxin (**57**) appear to only have anti-inflammatory activity, isoimperatorin (**55**) demonstrates only an analgesic effect. Columbianetin (**43**) inhibits the production of inflammatory cytokines induced by lipopolysaccharide (LPS), which is involved in the downregulation of nucleotide-binding and oligomerization domain 1 (NOD1)/NF-*κβ* pathways (Lu et al., 2018). The methanol extract from the root of *A. pubescens* have inhibitory activities on rat hind paw edema induced by carrageenan and on writhing induced by acetic acid in mouse, and the active principle was isolated and identified as osthol (Kosuge et al., 1985). The anti-inflammatory and analgesic constituents from *A. pubescens* appear to be related to the peripheral inhibition of inflammatory substances and to have influences on the central nervous system (Chen et al., 1995).

The essential oil from RAB (ERAB) has anti-inflammatory and analgesic effects (Fan et al., 2009). The effect of EAPR on inflammation is mediated by the inhibition of N-acylethanolamine-hydrolyzing acid amidase (NAAA) activity, which increases cellular endobioactor N-palmitoylethanolamine (PEA) levels while decreasing proinflammatory factor (Sun et al., 2011).

### Effects on the Central Nervous System

Brain aging is a neurodegenerative disease, which is incurable debilitating disorders characterized by structural and functional neuronal loss. The primary disease of brain aging is dementia, which includes VD, AD, and a mixed type of both (Wang et al., 2011a). Free radical damage and immune inflammatory mechanisms have been recognized in current research regarding the mechanisms of brain aging. Because of its large oxygen load, the brain becomes the most vulnerable target of oxygen free radicals, which can damage the central nervous system through a variety of processes, including causing the necrosis or apoptosis of nerve cells (Jin et al., 2003).

A water maze test on mice of a D-galactose-induced brain aging model was performed to study the ability of learning and memory (Jin et al., 2003). The authors found that RAB and its alcohol extract at 18 mg/kg*/*d can repair the membrane phospholipid structure in different parts of the mouse cerebral cortex and striatum, as well as improve the IL-2 content of aging model mice and resist free radicals and inflammatory damage, thereby improving the learning and memory ability of mice (Pei et al., 2005). From the perspective of inhibiting the apoptotic rate of brain tissue cells, the mechanism of RAB water decoction and its alcohol extract in retarding brain aging was revealed.

RAB and/or its water decoction (2 ml/d, *i.g.*) have an inhibitory effect on the inflammatory response of AD model rats induced by Human amyloid beta peptide 1–40 (A*β_1_*_-40_), thus improving the learning and memory ability of AD model rats (Zhu et al., 2009; Zhang and Du, 2010). The water decoction of RAB (1.08 mg/ml, *i.g.*) can improve the positioning and learning and memory ability of model rats, shortening the time to navigate the water maze (Du et al., 2009). CRAB (purity 95.8%) possess neuroprotective effects in A*β* damaging neuron (Yan et al., 2016). In addition, CRAB (purity 72.4%, 14.4 mg/kg, *i.g.*) can significantly protect amyloid precursor protein/presenilin-1 (APP/PS1) mice from neural damage, likely through improving Neurofilament tirplet M expression and reducing apoptotic cells in the brain of APP/PS1 transgenic mice (Jiao et al., 2014). Moreover, the water decoction of RAB (3.125 g/kg, *i.g.*) can inhibit the expression of malondialdehyde (MDA) in rat serum and increase the activity of Superoxide dismutase (SOD) in order to inhibit free radical damage in VD model rats (Weng et al., 2012). Finally, APR has a regulating effect on immune inflammatory injury in AD and VD rats, particularly for the treatment of VD (Wang et al., 2011a). Extracts, including 95% ethanol, petroleum ether, ethyl acetate, and petroleum ether-ethyl acetate extracts of RAB do not appear to be toxic to SH-SY5Y cells. All of these may increase cell viability and exert antioxidant activity induced by H_2_O_2_ in SH-SY5Y cells, whereas the neuroprotective effects of the petroleum ether-ethyl acetate extract have been shown to be the strongest (Hu et al., 2013a). Osthole (**17**) protects bone marrow-derived neural stem cells (BM-NSCs) from oxidative damage through the phosphatidylinositol 3 kinase (PI3K)/protein kinase B (Akt-1) pathway, and it has been shown to improve the inflammatory environment of neurodegenerative diseases and promote the survival rate of transplanted NSCs (Yan et al., 2017).

### Effects on the Cardiovascular System

*γ*-aminobutyric acid (**85**) (10 mg/kg, *i.v.*) isolated from the water extract of RAB (WRAB) showed that it can treat a variety of empirical cardiac arrhythmias, with effects on the action potential of rat neoventricular muscle (Li et al., 1989a). The alcohol extract of RAB (ARAB) and WRAB both have anti-angiogenic effects (Zou et al., 2008; Hu et al., 2013b). Osthole (**17**) isolated from ARAB inhibits angiogenesis *in vitro* more strongly than that in the ARAP, indicating that it may be the main antiangiogenic component of ARAB, and its mechanism may be related to the inhibition of human umbilical vein endothelial cell (HUVEC) proliferation, migration and tubule formation, the induction of HUVEC apoptosis, and the arrest of HUVEC cell cycle (Hu et al., 2013b). ARAB inhibits platelet aggregation and platelet thrombosis in circulating blood (Meng et al., 1988; Li et al., 1989b). Columbianetin (**43**), columbianetin acetate (**41**), columbianadin (**40**), osthol and columbianetin-*β*-D-glucopyranoside (**44**) also have inhibitory effects on rat platelet aggregation induced by ADP *in vitro* (Li et al., 1989b).

Pulmonary arterial hypertension (PAH) is a progressive cardiovascular-disease with high mortality lacking high-efficiency drug. Excitingly, osthole (**17**) extracted from RAB was observed to significantly restore 98 of 315 differential proteins significantly modified by PAH progression (Yao et al., 2018). What's more, osthole (**17**) extracted from the root of *A. pubescens* has been shown to suppress tracheal smooth muscle contraction, exerting a non-specific relaxant effect on the trachealis by inhibiting cAMP and cGMP phosphodiesterases (Teng et al., 1994). However, its dilative effect is dependent on endothelial integrity and NO production, and is mediated by the endothelial PI3K/Akt- endothelial NO synthase (eNOS)-NO pathway (Yao et al., 2013). These findings may provide candidates for a new pulmonary vasodilator for the therapy of PAH.

### Deworming Activity

RAB extracts using different solvents have quite different effects on treating ring worm. ARAB exhibited the best anthelmintic efficacy with 100% mortality of dactylogyrus and no death of the fish at the optimal anthelminthic concentration of 120 mg/L (Wang et al., 2011c; Xie et al., 2016). Osthole (**17**) can reach the 100% parasiticidal rate when the concentration was 1.6 mg/L, with no toxicity to fish at a dose up to 6.2 mg/L. As for scopoletin (**21**), 5 mg/L appears to represent a parasiticidal rate of 74.9% (Wang et al., 2011c; Wang et al., 2012).

### Others

Osthole (**17**) also shows osteopromotive effects on osteoblasts both *in vitro* and *in vivo*. Osthole (**17**) -mediated osteogenesis is related to the activation of the cAMP/cAMP response element-binding protein (CREB) signaling pathway and downstream osterix expression (Zhang et al., 2017). N-hexane and dichloromethane extracts of RAB show inhibitory effects on COX-1 and 5-LO. Linoleic acid appears to be the most active constituent in the extract, exerting COX-1 inhibition, and also has strong inhibitory activity on 5-LO, which is similar to osthole (**17**) (Liu et al., 1998b).

## Pharmacokinetic

So far, few pharmacokinetic studies on APR have been done, which mainly focus on coumarins. What's more, only medicinal plants *A. biserrata* have been studied. The main pharmacokinetic researches of the extracts or isolated compounds from APR that have been reported studies in animal are discussed below.

Four of the coumarins [columbianetin acetate (**41**), imperatorin (**54**), isoimperatorin (**55**), and osthole (**17**)] were rapidly absorbed, while the remaining three coumarins [psoralen (**51**), bergapten (**52**), and xanthotoxin (**57**)] were slowly absorbed in rat plasma after oral administration of APR extract (6.0 g/kg) (Chang et al., 2013). To assess the brain distributions and blood-brain barrier permeabilities of APR, a UPLC-MS/MS method was applied to the simultaneous determinations of the main coumarins in the rat cerebrospinal fluid (CSF) and brain after oral administration of APR extract (4 g/kg), including psoralen (**51**), xanthotoxin (**57**), bergapten (**52**), isoimperatorin (**55**), columbianetin (**43**), columbianetin acetate (**41**), columbianadin (**40**), oxypeucedanin hydrate (**56**), angelol B (**4**), osthole (**17**), meranzin hydrate (**20**), and nodakenetin (**69**). Most of the tested coumarins entered the rat CSF and brain quickly, and double-peak phenomena in concentration-time curves were similar to those of their plasma pharmacokinetics. Columbianetin (**43**) had the highest concentration in the CSF (C_max_: 485.36 ± 91.40 µg/L, AUC_0→∞_: 4142.82 ± 602.33 µg/L/h) and brain, while psoralen (**51**) and columbianetin acetate (**41**) had the largest percent of CSF/plasma and brain/plasma. (Yang et al., 2018). Under the administration mode and dose as above, columbianetin (**43**) was also easier to absorbed compound across caco-2 cell, and also had extremely highest plasma concentration (2000 ng/ml) *in vivo* (Yang et al., 2017). The pharmacokinetic properties of columbianetin (**43**) in rat after oral administration were characterized as rapid oral absorption, quick clearance and good absolute bioavailability. Columbianetin (**43**) showed dose proportionality over the dose range 5–20 mg/kg. The bioavailability of columbianetin (**43**) is independent of the doses studied (Luo et al., 2013).

The tissues distributions research after oral administration of APR extract (6.0 g/kg) to rat, four coumarins [columbianetin (**43**), columbianetin acetate, (**41**) osthole (**17)**, columbianadin (**40**)] were in the liver, followed by the ovary, uterus, kidney, lung, heart, spleen, and muscle (Ge et al., 2019). The AUC and C_max_ of bisabolangelone (**80**) after oral administrated pure bisabolangelone (**80**) are higher than those after oral administrated APR extract. Among them, there are significant differences on AUC between pure bisabolangelone (**80**) (7.5 mg/kg *i.g.*) and APR extract (equivalent to bisabolangelone (**80**) at dose of 7.5 mg/kg, *i.g.*). For tissue distribution, the amount of bisabolangelone (**80**) mainly appeared in heart, liver and spleen. Bisabolangelone (**80**) appearance in the brain also revealed that bisabolangelone (**80**) could pass the blood-brain barrier. (Ge et al., 2018).

A sensitive LC-MS/MS method has been validated to determine the concentration of columbianadin (**40**) in rat plasma after intravenous administration of columbianadin (**40**) (1, 2.5 and 5 mg/kg). The results show that the distribution half-life (T_1/2α_) were 0.027 0.016, 0.060 0.065, and 0.028 0.023 h, and the elimination half-lives (T_1/2β_) were 0.58 0.20, 0.52 0.25, and 0.52 0.22 h (Chang et al., 2014). After oral administration of pure columbianadin (**40**), approximately 0.12% columbianadin (**40**) was transformed into columbianetin (**43**). Oral administration of pure columbianadin (**40**) may be more beneficial for the clinical efficacy of columbianadin (**40**) (25 mg/kg) than APR extract (3.74 g/kg). Pure columbianadin (**40**) group: T_max_: 3.03 ± 1.87 h, C_max_: 1.82 ± 0.64 ng/ml, AUC_(0-tn)_: 1.05 ± 1.02 ng/ml/h; APR extract group: T_max_: 0.55 ± 0.33 h, C_max_: 13.33 ± 25.37 ng/ml, AUC_(0-tn)_: 28.80 ± 41.46 ng/ml/h (Li et al., 2018).

Columbianetin acetate (**41**) and columbianetin-*β*-D-glucopyranoside (**44**, CBG) were rapidly and widely distributed in rats, and eliminated rapidly from plasma. Columbianetin acetate (**41**) could be metabolized into columbianetin (**43**) *in vivo*. Absolute bioavailability of pure columbianetin acetate (**41**) is 7.0 ± 4.3%. Other co-existing ingredients in APR extract could increase the concentration of its metabolite columbianetin (**43**) in plasma and this was caused by CBG (**44**). Cumulative excretion of columbianetin acetate (**41**) in urine accounted for 0.0109 ± 0.0067% of total dosage. The cumulative amounts of columbianetin acetate (**41**) in the feces present 9.32 ± 6.63% of the total dose. Columbianetin acetate (**41**) was mainly excreted in the feces (Jiao et al., 2015). Besides, CBG (**44**) also could be catabolized into its active metabolite columbianetin (**43**) *in vivo*. The absolute bioavailability of CBG (**44**) was 5.63 ± 4.42%. The other co-existing constituents from the APR ethanol extract could enhance the absorption of CBG (**44**). CBG (**44**) and columbianetin (**43**) were rapidly and broadly distributed in the stomach, ovary, kidney, liver, spleen, lung, muscles, heart and brain. Higher levels of accumulation of CBG (**44**) and columbianetin (**43**) were detected in the ovary and kidney tissues. Eight metabolites of CBG (**44**) were tentatively identified in blood, urine, bile and faeces of rats after oral administration of pure CBG (**44**). It was also found that CBG (**44**) and columbianetin (**43**) were mainly excreted through the faecal route (Zhang et al., 2018).

## Toxicology

In ancient and modern books of traditional Chinese medicine including with APR, there was no record of toxicity. The records of toxicity also have not been reported in clinical applications. So far, only a few toxicity tests have been conducted in animals.

In acute toxicity experiments through oral administration, mice of the Kunming species appeared to have cyanosis, agitated activity, and a quickened respiration after 10 min of taking water decoction of APR, and several mice died because of respiratory failure in serious cases. The LD_50_ of mice (*i.g.*) was determined to be 7.35 ± 0.62g**/**kg (Duan et al., 2002). In subacute toxicity testing, WAPR has a certain nephrotoxicity, potentially inducing kidney injury through inhibiting organic anion transporters 1 (Oats1), Oat2, Oat3 (Chen et al., 2015). WAPR also can cause obvious organ toxicity to juvenile zebrafish, such as yolk sac swelling, deformation, black, pericardial edema, and bleeding, with a minimum lethal dose of 100 μg/ml at 3 days after fertilization in healthy zebrafish embryos (Chen et al., 2016). In a 90-days toxicity experiment by mouth, Wistar rats and hybrid dogs were selected. Among them, rats appeared to have cyanosis, agitated activity, and quicken respiration after 10 min of taking APR capsule; however, toxicity symptoms in the rats disappeared after 30 days of taking WAPR. At higher doses of WAPR, the rats grew slower, and pathology inspection revealed gaseous distention in the stomach and edema of mucous membrane. While there were myeline bodies in the liver cells of some rats and dogs, it appeared to be reversible. No differences in the blood and biochemistry indexes of rats and dogs were observed (Duan et al., 2002).

## Quality Control

APR has special aromatous smell, bitter and spicy taste, and makes feeling slightly numbness of tongue. It is traditionally thought that APR with thick and fat branches, strong aroma is of good quality. From the phytochemistry and the pharmacological activity discussed above, coumarins are considered the most important constituents, demonstrating a wide range of pharmacological activities. In Chinese Pharmacopoeia (2015 edition), APR is calculated by dry product, containing osthole (**17**) at not less than 0.50% and columbianadin (**40**) at not less than 0.080%. What's more, quality control items for APR in multi-national Pharmacopoeias are listed in **Table 5**.

**Table 5:** Quality control items for APR in multi-national Pharmacopoeias.

Pharmacopoeia	Identification	Water detection or Loss on drying	Ash detection		Determination of content	
			Total ash	Acid insoluble ash	Marker components	Content requirements
Chinese Pharmacopoeia (version 2015)	Fragments identification and TLC	≤10%	≤8%	≤3%	Osthole and columbianadin	The total content≥0.080% (dried drug).
British Pharmacopoeia (version BP2017)	Microscopic examination and TLC (Reference solution: (Z)-Ligustilide, Osthole and Imperatorin)	≤10%	≤8.0%	≤3.0%	Osthole	The total content≥0.50% (dried drug).
European Pharmacopoeia (version EP9.3)	Microscopic examination and TLC (Reference solution: (Z)-Ligustilide, Osthole and Imperatorin)	≤10%	≤8.0%	≤3.0%	Osthole	The total content≥0.50% (dried drug).

APR, Angelicae Pubescentis Radix.

APR primarily contains coumarins and volatile oils as the active ingredients (Zhang et al., 2007). Near IR spectroscopy (NIRs) became a new analysis technique for the quantitative analysis of Chinese medicinal materials because of its rapid analysis speed and no need for sample pretreatment. NIRs also can achieve quantitative analysis for the quality control of osthole (**17**) and columbianadin (**40**) as indicators in APR, except for HPLC (Zhan et al., 2017). Modern pharmacological research has shown that ferulic acid (**75**) is also the main effective component, providing the effect of activating the blood. However, its content has not been used as an index in Chinese Pharmacopoeia, which is not conducive to the quality control of APR. Therefore, the method for the determination of ferulic acid (**75**) content in APR was established by reverse HPLC (Fang, 2013). Bisabolangelone (**80**), adenosine (**78**), and 30 coumarins in APR were identified by UPLC-PAD-Q-TOF-MS. The developed method could successfully be used to differentiate samples from different regions and could be a helpful tool for detection and confirmation of the quality of TCM (Ge et al., 2014). However, one or several chemical constituents are not the only effective parts of APR. The HPLC fingerprint of APR from different producing areas was constructed with 21 common chromatographic peaks, and the content of columbianetin (**43**), osthole (**17**), isoimperatorin (**55**), and columbianadin (**40**) were determined in APR simultaneously (Wang et al., 2018b). The GC fingerprint of APR was also established (Jin et al., 2009).

## Conclusion and Future Perspectives

A significant breakthrough has been made in the last several decades in the areas of phytochemistry and pharmacology of APR. Research on phytochemistry indicated that coumarins and essential oil compounds might be the major active constituents. Crude extracts and pure compounds from the root have been shown to possess multiple biological activities, particularly analgesic and anti-inflammatory actions, as well as effects on the central nervous system. However, further study is still urgently needed to gain a better understanding of APR and its clinical use.

Firstly, 87 compounds, mainly coumarins, have been separated and identified, and more than 100 volatile components have been analyzed by GC-MS, but they were mainly separated from root of *A. biserrata*. Therefore, in further research on the phytochemistry of this plant, more attention should be focused on the others parts, such as rhizome, fruit. Besides, the water-soluble part of APR should be a major focus, because decoction is the main method of administration in the TCM clinic. The majority of pharmacological studies of APR have been conducted using crude and poorly characterized extracts. Thus, bioactivity-guided isolation strategies could be used to study the chemicals underlying the pharmacological activity. To illustrate the scientific significance of medicines' using, network pharmacology should be used to predict drug targets and the possible molecular mechanisms involved. Then experiments should be further conducted to prove whether the targets and signal path works.

Secondly, studies on pharmacological effects and its mechanisms mainly focus on several coumarins, and the inherent relations between them and the mechanism of treatment of disease have not been explicit enough. More relationships between the chemical composition and pharmacological activity should be established to further explain the principle of disease treatment. Moreover, the pathways of their distribution, absorption, metabolism, and excretion need to be further clarified with pharmacokinetic studies.

Thirdly, records of toxicity were rarely recorded in ancient clinical applications; however, modern pharmacological research has related acute toxicity in animal experiments. Meanwhile, it should be noticed that most pharmacological studies on APR have only been conducted in animal models, cell models, and other *in vitro* experiments. Therefore, comprehensive placebo-controlled and double-blind clinical trials should be undertaken to provide remarkable evidence for these positive findings on the efficacy of APR in the future. In addition, the exact mechanisms of many medicinal properties of this herb still remain vague to date; thus, additional studies to better identify the functions and molecular targets seem to be necessary.

Next, the result of the research on plant resources found that *A. biserrata* had been mistaken as *A. pubescens* in China. At present, *A. pubescens* is only a medicinal plant for local use with a few researches, and the specific difference between the two medicinal plants in clinical use have not been mentioned. In order to standardize the use of traditional Chinese medicine, it is necessary to study the specific differences in the clinical use and pharmacological effect of the two medicinal plants.

Lastly, the natural resources of APR are limited, and the demand is increasing year by year. Since April 2018, the State Administration of Traditional Chinese Medicine of the People's Republic of China issued a list of Chinese classical prescriptions (first batch), in which six prescriptions contain APR. This means that the needs of APR will continue to grow. In the course of the determination of the content, our laboratory collected some unqualified cultivation products, so we considered that it might be caused by irregular planting methods. So, the safe, high quality and high efficiency planting technology of this unique living plant needs to be further studied in order to guide the production of TCM.

In summary, APR has been successfully used in clinical practice to treat rheumatic disease as an anodyne for thousands of years. Indeed, modern pharmacological studies have shown that it has effective analgesic and anti-inflammatory actions, as well as effects on the central nervous system. The linkage between traditional uses and modern scientific studies, safety, and efficacy were done on this herb ensuring its clinical use and application.

## Author Contributions

YL and HW searched the literature, collected the data, and drafted the manuscript. YL and ZW contributed to analysis and manuscript preparation. XY, XZ, and HL helped in checking the chemical structures and formula. YL and LT downloaded the documents and made classification. ZW and LT contributed comments for a version of the manuscript. All authors read and approved the final manuscript.

## Funding

The work was supported by grants from the National Key Research and Development Plan (2018YFC1707106), the Key Research and Development Plan of Shandong Province in 2018 (No. 2018CXGC1305) and National Science and Technology Major Projects for “Major New Drugs Innovation and Development” (2019ZX09201005). Basic scientific research project of the Institute of Chinese Materia Medica, China Academy of Chinese Medical Science (ZXKT17014).

## Conflict of Interest

The authors declare that the research was conducted in the absence of any commercial or financial relationships that could be construed as a potential conflict of interest.
